# Investigating the Effects of Pulmonary Rehabilitation via Social Media Versus Brochures on General Health in Nonhospitalized COVID-19 Patients: A Randomized Controlled Trial

**DOI:** 10.1155/cjid/9676407

**Published:** 2025-09-05

**Authors:** Sheida Shojaei, Hamid Reza Farpour, Rezvan Ghaderpanah, Amin Sayyadi

**Affiliations:** ^1^Student Research Committee, Department of Physical Medicine and Rehabilitation, Faculty of Medicine, Shiraz University of Medical Sciences, Shiraz, Iran; ^2^Shiraz Geriatric Research Center, School of Medicine, Shiraz University of Medical Sciences, Shiraz, Iran; ^3^Orthopedic & Rehabilitation Research Center, Department of Physical Medicine and Rehabilitation, Shiraz University of Medical Sciences, Shiraz, Iran; ^4^School of Medicine, Kerman University of Medical Sciences, Kerman, Iran

**Keywords:** breathing exercise, COVID-19, pulmonary rehabilitation, social media

## Abstract

**Background:** The global impact of COVID-19 has presented challenges to health systems, affecting both physical and psychological well-being. Nonhospitalized patients, constituting the majority, can benefit from pulmonary rehabilitation through breathing exercises. This study aims to evaluate the effect of breathing exercises delivered via social media on the general health of nonhospitalized individuals with mild COVID-19.

**Methods:** In a randomized controlled trial conducted at university hospitals, ninety patients aged 18–65 without prior respiratory or other specified disorders were included. The intervention group learned breathing exercises from online videos on Instagram, while the control group received brochures. Both groups practiced 6 days a week for three sessions a day. Results were evaluated using General Health Questionnaire (GHQ-28), Patient Health Questionnaire (PHQ-15), Barthel Index (BI), and Visual Analogue Scale (VAS).

**Results:** Both groups improved significantly in terms of GHQ-28 and all of its subscales, PHQ-15, and VAS (*p*-value < 0.05), but none achieved significant improvements in BI (*p*-value > 0.05). The effect size was large in all criteria except for GHQ-28 depression symptoms in the Online group (Cohen's d = 0.347; 95% CI 0.103, 0.59), and GHQ-28 anxiety/insomnia (Cohen's d = 0.794; 95% CI 0.494, 1.095) and GHQ-28 depression symptoms (Cohen's d = 0.453; 95% CI 0.135, 0.771) in the Brochure group. The differences between the Online and Brochure groups were insignificant in all assessed criteria (*p*-value: GHQ-28 = 0.231; PHQ-15 = 0.166; VAS = 0.385; BI = 0.165).

**Conclusions:** Breathing exercises through social media and brochures significantly improve physical and psychological aspects in mild COVID-19 patients. While comparison with face-to-face interventions was not possible, the results are promising, encouraging physicians to consider this intervention, especially for underdeveloped countries and patients facing access barriers.

**Trial Registration:** Iranian Registry of Clinical Trials (IRCT): IRCT20201204049600N1

## 1. Introduction

The appearance of coronavirus disease 2019 (COVID-19), as a cause of severe respiratory illness, has posed new challenges to public health systems worldwide. In addition to its physical complications, COVID-19 has also caused substantial mental health problems. Social isolation regulations, implemented to stop the spread of the virus, along with uncertainty about what lies ahead, have resulted in depression, anxiety, sleep disturbances, and social dysfunction among the general population [1, 2].

Approximately 80% of COVID-19 cases are asymptomatic or present with mild symptoms, and most patients recover without hospitalization [3]. However, even mild cases can suffer from undesirable physical and psychological effects that reduce quality of life. We can use nonpharmacologic interventions such as pulmonary rehabilitation to mitigate these issues. A multidisciplinary rehabilitation team typically supervises pulmonary rehabilitation, encompassing physical therapy, patient education, psychological intervention, airway clearance, and breathing exercises. Studies have demonstrated that these interventions have the potential to alleviate physical symptoms like dyspnea and leg discomfort, and psychological problems such as sleep disturbances and impaired daily functioning [4–6].

Breathing exercises such as diaphragmatic and pursed-lip breathing are widely used techniques in pulmonary rehabilitation that reduce the respiratory rate and extend expiratory time, thus improving lung hyperinflation and oxygenation [7]. Monitoring these programs is essential, but during social distancing and in mild COVID-19 cases, modified breathing exercises combined with online patient education can be appropriate alternatives because many patients avoid health centers due to fears of infection, quarantine policies, or limited access [8, 9].

Several studies have investigated the feasibility of social media in delivering rehabilitative interventions in a variety of conditions, such as cardiac disorders [10] and breast cancer [11], and have shown promising results. Although multiple studies have explored the potential of social media for pulmonary rehabilitation, most have focused on patients with chronic obstructive pulmonary disease (COPD) [12, 13], leaving a gap in the literature regarding its use in patients with mild COVID-19.

To the best of our knowledge, no study has investigated the impacts of social media-delivered rehabilitation on COVID-19 patients. To address these gaps, we carried out a randomized controlled trial to compare the effects of breathing exercises administered via social media with those provided via brochures on the general health of nonhospitalized patients with mild COVID-19. This study aims to determine whether telerehabilitation through social media platforms is effective during the COVID-19 recovery period.

## 2. Methods

### 2.1. Patients and Setting

We conducted a randomized, multicenter clinical trial with a parallel group to investigate the effects of breathing exercises on the general health of patients diagnosed with mild COVID-19. Patients were recruited from three university hospitals (Chamran Hospital, Faghihi Hospital, and Ali Asghar Hospital of the Shiraz University of Medical Sciences, Shiraz, Iran). This study followed the CONSORT reporting guideline (Supporting Information 1) for randomized clinical trials.

The inclusion criteria for this study encompass the following: (1) age between 18 and 65 years, (2) having a positive polymerase chain reaction (PCR) for COVID-19, (3) no or mild lung involvement on chest x-ray or CT, (4) no need for hospitalization and the ability to care for oneself, (5) stability of clinical symptoms, and (6) ability to understand and learn through online learning. The exclusion criteria for this study entail the following: (1) the presence or history of other respiratory diseases like asthma and COPD; (2) advanced cardiac disorders such as heart failure and ischemic heart disease (IHD); (3) a history of liver, kidney, or thyroid disease; (4) prior hospitalization in the intensive care unit (ICU) due to coronary artery disease (CAD); (5) a history of lower motor neuron diseases, including peripheral neuropathy, neuromuscular disorders, and myopathy; (6) a history of hypertension (HTN) or diabetes mellitus (DM); (7) a history of upper motor neuron diseases, such as stroke or multiple sclerosis (MS); (8) insufficient literature or lack of access to the internet; and (9) the absence of informed consent.

### 2.2. Randomization and Blinding

After recruiting the patients, we assessed them based on the inclusion and exclusion criteria and excluded those who were ineligible. Before randomization, the demographic characteristics and baseline data of the included patients were assessed. Two identical groups of patients were randomly assigned using the block randomization method with two permutations, AB and BA. In this way, numbers were randomly selected from the table of random numbers. If the numbers were between zero and four, they were assigned to block AB; if they were five to nine, they were assigned to block BA. This resulted in two equal lists of individuals. We randomly formed two groups: the Intervention (Online) and the Control (Brochure) groups. For concealment, this random assignment method was given to another person who did not know about the research process, and the questionnaires were filled out by this person who was unaware of the allocation of patients in the groups. All assessors were blinded to group assignments, hypotheses, and intervention details. The patients could not be blinded for the allocation.

### 2.3. Intervention

After being informed of the educational program procedure, patients participated in the study and gave informed consent. Demographic and baseline data, General Health Questionnaire (GHQ-28), Patient Health Questionnaire (PHQ-15), Visual Analogue Scale (VAS), and Barthel Index (BI) were completed. The Online group assumed that they would learn the breathing exercises online through videos uploaded to Instagram. Therefore, the patients were sent the link to the desired page, and patients' access to the page was ensured. On the other hand, instructions and brochures were sent to the Brochure group, ensuring accessibility for this group as well. During the program, all the participants could contact the physician in case of any problems. The groups were supervised twice a week to reduce the number of dropouts and monitor the exercise regimen. The exercise techniques and the respective number of repetitions were described in detail in videos and brochures. Breathing exercises consisted of diaphragmatic/belly breathing, pursed-lip breathing, sniffing, shoulder shrug, breathing hold, and active cycle breathing (for airway clearance and cough stimulation). The schedule for both groups was six times per week, three sessions per day, and each session five times, lasting approximately 10–20 min. After 4 weeks, all the questionnaires were completed again.

### 2.4. Assessment of the Outcomes

#### 2.4.1. GHQ-28

GHQ-28 is a self-reported GHQ with four subscales: somatic symptoms, anxiety, insomnia, social disorders, and depression. It is used for detecting mental disorders and consists of 28 items, each of which is scored on a 4-point scale to determine the intensity of the encountered complaints or symptoms ranging from 0 (Not at all) to 3 (Much more than usual). The total score ranges from 0 to 84, with higher scores indicating greater distress [14].

#### 2.4.2. PHQ-15

PHQ-15 was developed by Kroenke et al. [15] and measures subjective somatic symptoms. It consists of 15 questions with three response options: (0) *not bothered at all*, (1) *slightly distressed*, and (2) *very distressed*. Symptom severity is classified based on the total score as: *minimal* (0–4), *mild* (5–9), moderate (10–14), and severe (≥ 15).

#### 2.4.3. BI

It assesses a person's physical condition and ability to perform daily activities, including eating, bathing, mobility, stair climbing, dressing, and personal hygiene. This questionnaire consists of 10 sections, each with its corresponding score. A total of 20 points are given for the entire questionnaire [16].

#### 2.4.4. VAS

It is a common and popular tool for assessing pain intensity. Patients answer the questions by selecting a number from 0 to 10, where 0 indicates no pain and 10 represents the most intense pain experienced [17].

### 2.5. Sample Size and Statistical Analysis

Using Power SCC software, a power of 80%, a significance level of 0.05, and an anticipated dropout rate of 10%, we recruited 90 patients. Quantitative data were summarized using measures of central tendency and dispersion, including the mean and standard deviation. We used the chi-square test to compare categorical variables between groups, such as smoking, alcohol consumption, sex, marital status, education, number of children, and employment status. Independent *t*-tests were performed to assess between-group differences in continuous variables, including age, BMI, GHQ-28, PHQ-15, BI, and VAS scores. Furthermore, we applied paired *t*-tests to compare pre- and postintervention scores within groups. Cohen's d was used to determine the effect size. Data were analyzed using R (version 4.1.3) within RStudio (version 2024.04.2 + 764), and a *p*-value of less than 0.05 was considered statistically significant.

### 2.6. Ethical Considerations and Trial Registration

The study protocol adhered to the principles of the Declaration of Helsinki and was approved by the Ethics Committee of Shiraz University of Medical Sciences, Shiraz, Iran (approval number: IR.SUMS.MED.REC.1399.433). Written and verbal informed consent was obtained from all participants prior to enrollment. Data confidentiality was guaranteed.

## 3. Results

**Figure 1:**
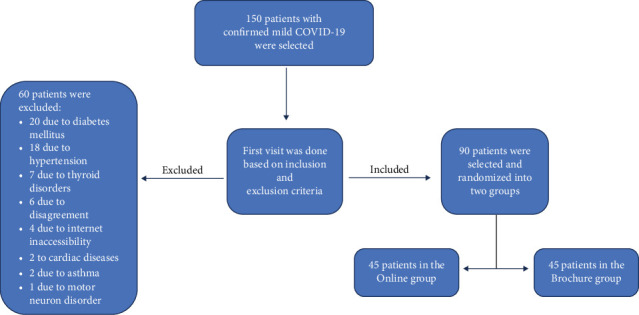
Consort flowchart.

A total of 150 individuals with mild COVID-19 symptoms were assessed to participate in the study. Of these, 90 patients met the inclusion criteria, were randomly assigned to one of two groups, and participated in the intervention for four weeks (Figure 1).

The mean age and BMI of the study population were 34.81 ± 9.2 years and 25.32 ± 3.06 kg/m^2^, respectively. Of the 90 participants, 52 (57.8%) were female. There were no statistically significant differences (*p*-value > 0.05) between the two groups in terms of age, sex, marital status, education, number of children, employment status, BMI, smoking, and alcohol consumption (Table 1).

### 3.1. GHQ-28

**Figure 2:**
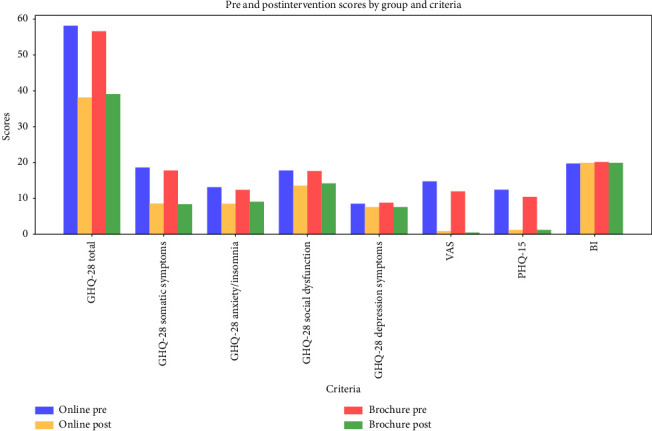
Comparison of pre- and posteducation scores between the two groups; GHQ: General Health Questionnaire; VAS: visual analogue scale; PHQ: Physical Health Questionnaire; BI: Barthel Index.

Both the Online and Brochure groups achieved significant improvements in total score and all the subscales of GHQ-28 (*p*-value < 0.05). In the Online group, total score (Cohen's d = 2.05; 95% CI = 1.5, 2.6) and all the subscales including somatic symptoms (Cohen's d = 2.863; 95% CI = 1.799, 3.928), anxiety/insomnia (Cohen's d = 0.743, 1.542; 95% CI = 0.743, 1.542), and social dysfunction (Cohen's d = 1.273; 95% CI = 0.786, 1.761) had large effect sizes; however, depression symptoms subscale showed a small effect size (0.347 95% CI = 0.103, 0.59). Using a Brochure resulted in large effects on GHQ-28 total score (Cohen's d = 1.766; 95% CI = 1.246, 2.287), somatic symptoms (Cohen's d = 3.043; 95% CI = 2.082, 4.003), and social dysfunction (Cohen's d = 1.43; 95% CI = 0.885, 1.974), but the effect on anxiety/insomnia was moderate (Cohen's d = 0.794; 95% CI = 0.494, 1.095) and the effect on depression symptoms was small (Cohen's d = 0.453; 95% CI = 0.135, 0.771) (Table 2, Figure 2).

The differences between the Online and Brochure groups were not statistically significant for the GHQ-28 total score or any of its subscales (*p*-value > 0.05). However, the Online group demonstrated better performance in improving the GHQ-28 total score and all subscales, except for depression symptoms. The effect size was negligible or small for the GHQ-28 total score and all of its subscales (Cohen's d < 0.5) (Table 2, Figure 2).

### 3.2. VAS

Both the online and brochure-based educational interventions resulted in significant improvements with large effect sizes in each group (Online: *p*-value < 0.001, Cohen's d = 1.353, 95% CI = 0.837 1.869; Brochure: *p*-value < 0.001, Cohen's d = 1.609, 95% CI = 1.018 2.201). The Online group demonstrated a greater improvement compared to the Brochure group, but the difference was not statistically significant (*p*-value = 0.307, Cohen's d = −0.193, 95% CI = −0.586, 0.199) (Table 2, Figure 2).

### 3.3. PHQ-15

Both the Online and Brochure groups achieved significant improvements in PHQ-15 scores following educational interventions with large effect sizes (Online: *p*-value < 0.001, Cohen's d = 2.528, 95% CI = 1.481, 3.574; Brochure: *p*-value < 0.001, Cohen's d = 1.943, 95% CI = 1.058, 2.827). Although the Online group showed a greater improvement than the Brochure group, the difference between them was not statistically significant (*p*-value = 0.211, Cohen's d = −0.352, 95% CI = −0.917, 0.213) (Table 2, Figure 2).

### 3.4. BI

BI did not change significantly in either the Online or Brochure groups (Online: *p*-value = 0.254, Cohen's d = −0.165, 95% CI = −0.45, 0.121; Brochure: *p*-value = 0.323, Cohen's d = −0.211, 95% CI = −0.634, 0.213). Additionally, the difference between the two groups was not significant (*p*-value = 0.334, Cohen's d = 0.206, 95% CI = −0.214, 0.626) (Table 2, Figure 2).

## 4. Discussion

This study investigated the effect of breathing exercises delivered through online education on the general health of patients with mild COVID-19 using GHQ-28, VAS, PHQ-15, and BI as outcome measures. Both the Online and Brochure groups showed significant improvements in GHQ-28, PHQ-15, and VAS scores (*p*-value < 0.05); in all cases, the effect size was large (Cohen's d > 0.8), except for GHQ-28 depression symptoms in the Online group (Cohen's d = 0.347; 95% CI 0.103, 0.59), and GHQ-28 anxiety/insomnia (Cohen's d = 0.794; 95% CI 0.494, 1.095) and GHQ-28 depression symptoms (Cohen's d = 0.453; 95% CI 0.135, 0.771) in the Brochure group. Although the Online group generally outperformed the Brochure group in terms of the difference between pre- and posteducation scores, the differences between them were not statistically significant (*p*-value > 0.05) in any criteria. Both methods efficiently improved the general health of mild COVID-19 patients. In contrast to GHQ-28, PHQ-15, and VAS, none of the groups could achieve significant changes in BI (*p*-value > 0.05).

Our results show the efficacy of telerehabilitation delivered via social media. While telerehabilitation is well-studied, evidence specifically involving social media is scarce. Dorje et al. [10] employed this method in 312 patients who had undergone previous percutaneous coronary intervention. They assigned them into two groups: an intervention group that received comprehensive cardiac rehabilitation and secondary prevention via social media, and a control group that received standard outpatient follow-up. The intervention group demonstrated significantly greater improvements in 6-min walk distance (6MWD) at both 2 months (adjusted mean difference: 20.64 m; 95% CI 7.50, 33.77; *p*-value = 0.034) and 6 months (adjusted mean difference: 22.29 m; 95% CI 8.19, 36.38; *p*-value = 0.027). They concluded that their approach is an effective, accessible, and easy-to-use cardiac rehabilitation service.

Rouzfarakh et al. [18] conducted an RCT comparing rehabilitation delivered via social media to traditional methods (brochures and face-to-face education) in 60 burn patients. At one and 2 months, social media-based education significantly improved the overall quality of life and all subscales of the Burn Specific Health Scale–Brief, except for body image (*p*-value = 0.550) and skin sensitivity (*p*-value = 0.333). They believed this method enhances the quality of life and can be an effective educational and follow-up tool for burn patients.

Li et al. [12] allocated 140 patients into three groups: social media supervision at home, maintenance at the hospital, and usual care. Both intervention groups demonstrated continuous clinical improvements in 6MWD, COPD Assessment Test (CAT), and modified Medical Research Council scale (mMRC) compared to usual care (*p*-value < 0.001). There was no significant difference between the two intervention groups (*p*-value > 0.05). Multivariate analysis revealed that both interventions were independent predictors of reduced AECOPD risk (home-based maintenance: incidence rate ratio 0.712; 95%CI 0.595, 0.841; *p*-value < 0.001 vs. hospital-based maintenance: incidence rate ratio 0.799; 95% CI 0.683, 0.927; *p*-value = 0.002).

Our study also revealed that brochures can effectively improve the general health of mild COVID-19 patients. This aligns with findings by Yilmaz et al. [19], who assessed a home exercise program with and without physiotherapist instruction in 80 patients with knee osteoarthritis. Both groups showed significant postintervention improvements in range of motion (ROM), VAS, quadriceps and hamstring muscles strength, Western Ontario and McMaster Universities Osteoarthritis Index (WOMAC), and Short Form Health Survey (SF-36) scores (*p*-value < 0.05).

Although social media generally resulted in better improvements than brochures in our patients, the differences were not significant. While direct comparisons between these methods are scarce, some studies have explored similar platforms. Pehlivan et al. [20] conducted an RCT evaluating postdischarge telerehabilitation practices in COVID-19 patients. They included two groups: the intervention group, which received breathing and ROM exercises, active cycle of breathing technique, and aerobic training 3 days per week for 6 weeks via live videoconferences; the control group, which received the same content via brochures. The intervention group achieved significant improvements in mMRC dyspnea score for dyspnea (*p*-value = 0.035), 30-s sit-to-stand test (*p*-value = 0.005), five sit-to-stand time subtest of short physical performance battery (*p*-value = 0.039), and Saint George Respiratory Questionnaire (*p*-value < 0.05). In contrast, the control group showed significant improvements only in pain scores (*p*-value = 0.039). The differences between groups were significant for the Saint George Respiratory Questionnaire (*p*-value = 0.035) and the total score (*p*-value = 0.042). The authors concluded that telerehabilitation with less technical equipment can be considered a suitable alternative for rehabilitation in COVID-19 patients, thereby enhancing quality of life and symptomatic status. This is likely due to the live, guided nature of video sessions led by physiotherapists.

Another study by Sunthornsup et al. [21] compared brochures and videos for educating 100 patients on disease-related knowledge. They measured the knowledge scores before (T0), immediately after (T1), and 4 weeks postintervention (T2). Both groups demonstrated significant improvements throughout the experiment (brochure group: 72.7 ± 20.3% vs. 51.1 ± 24.7%, *p*-value < 0.001; video group: 78.3 ± 15.7% vs. 56.1 ± 21.9%, *p*-value < 0.001). When comparing the improvement from T0 to T1, the video group had significantly higher mean score at T1 compared to brochure group (video group: 86.7 ± 12.9% vs. brochure group: 76.0 ± 21.4%, *p*-value = 0.003), but no significant difference was observed between T1 and T2 (brochure group: −4.7 ± 13.3% vs. video group: −8.5 ± 11.0%, *p*-value = 0.152). These two studies suggest that videos might offer certain advantages over brochures; however, live performance might be necessary to maximize this superiority. This aligns with our results, which show that offline videos do not have a significant advantage over brochures. We emphasize the need for further research to compare live versus pre-recorded video education to better clarify this matter.

Another finding was that none of the interventions significantly affected BI scores. Several factors can be noted. First, BI measures physical condition and daily activities, and our mild COVID-19 patients were nonhospitalized with high baseline levels of independence (Online group: 19.78 ± 1.06; Brochure group: 19.98 ± 0.15), limiting the room for measurable improvement. Second, pulmonary rehabilitation is primarily focused on improving respiratory function, which is not usually reflected in BI. Finally, BI mainly assesses activities such as eating, bathing, and mobility, which are typically unaffected in mild COVID-19; thus, BI may not be able to detect subtle improvements in overall health or respiratory function.

Although hospital-based pulmonary rehabilitation offers clear benefits, its application is often restricted by high costs, unequal access, and geographical barriers. Telerehabilitation, especially when delivered via social media, offers a practical alternative, and recent studies have focused more on this new opportunity. A 2021 Cochrane metanalysis [22] showed that social media interventions effectively improve physical activity and well-being with no reported adverse effects, supporting their efficacy and safety.

Despite our results indicating a nonsignificant superiority of telerehabilitation over brochures, these two hold unique advantages. Telerehabilitation is cost-effective for both healthcare providers and patients, time-saving, and accessible from various locations and for different issues [23]. Additionally, patients can clearly learn the exercises through video clips [24]. On the other hand, brochures are effective tools widely utilized in society, especially by individuals without digital skills.

Although the COVID-19 pandemic has highlighted the massive potential of online education [25], certain obstacles remain, particularly in developing countries. A systematic review [26] identified slow speed and limited internet coverage, legal concerns, and skepticism as common challenges. Other issues include high costs, data privacy, and limited digital proficiency. Furthermore, concerns exist regarding reduced human interaction and the need for patient-specific customization, which may increase costs [24]. Moreover, most studies are done in high-income countries with established services, while middle- and low-income countries face greater challenges in providing such services. Further research is needed in these regions to address their unique constraints.

This study contributes to the growing body of evidence supporting the efficacy of pulmonary rehabilitation, particularly in the form of breathing exercises and telerehabilitation, for the recovery of COVID-19 patients. Given the clear benefits of telerehabilitation, policymakers may need to consider using this affordable and accessible method as an alternative to face-to-face rehabilitation, which could substantially reduce morbidity—a significant issue in developing countries. Debates continue regarding the effectiveness of rehabilitative interventions via social media versus brochures, as each has its unique features. Further research, utilizing improved study designs and more comprehensive evaluation criteria, is necessary to elucidate the lesser-known aspects of this topic.

### 4.1. Limitations

An important limitation was that blinding participants was not feasible due to the nature of the interventions. Another issue was that we did not include face-to-face or no-treatment control groups, limiting the scope of comparisons. Repeated-measures ANOVA is generally recommended for evaluating time effects and the group × time interaction; however, we used paired and independent *t*-tests due to the presence of only two time points and the specific objectives of our study. Although this approach is statistically valid and consistent with prior literature [27, 28], we acknowledge that this represents a minor methodological limitation. Furthermore, we administered outcome measures in a fixed sequence to ensure consistency across participants and practicality, but this may have introduced potential bias. The results may not be generalizable to all COVID-19 patients, as we included only individuals with mild symptoms. In addition, the follow-up period was only 4 weeks.

### 4.2. Conclusion

Pulmonary rehabilitation, including breathing exercises, can be effectively delivered through social media and brochures, and has been shown to significantly improve physical and psychological outcomes in patients with mild COVID-19, including general health, pain, and physical symptoms. Despite the study's limitations, the results are promising. We encourage physicians to consider these interventions in future therapies, especially in underdeveloped countries and for patients with specific socioeconomic or geographical barriers. Future studies should involve a more diverse patient population, extended follow-up periods, and more appropriate control groups to develop and improve COVID-19 pulmonary rehabilitation strategies.

## Figures and Tables

**Table 1:** Baseline characteristics of the Online and Brochure groups.

Characteristic		Online group	Brochure group	*p*-value
Age (years: mean ± SD)		32.96 ± 8.20	36.67 ± 9.85	0.055

BMI (kg/m^2^: mean ± SD)		25.25 ± 3.29	25.40 ± 2.85	0.814

Smoking (*N* (%))	Yes	11 (24.44%)	10 (22.22%)	0.803
	No	34 (75.56%)	35 (77.78%)	

Alcohol consumption (*N* (%))	Yes	14 (31.11%)	7 (15.56%)	0.081
	No	31 (68.89%)	38 (84.44%)	

Sex (*N* (%))	Male	16 (35.56%)	22 (48.89%)	0.200
	Female	29 (64.44%)	23 (51.11%)	

Marital status (*N* (%))	Married	26 (57.78%)	28 (62.22%)	0.529
	Single	17 (37.78%)	13 (28.89%)	
	Other	2 (4.44%)	4 (8.89%)	

Education (*N* (%))	Academic	28 (62.22%)	26 (57.78%)	0.667
	Non-academic	17 (37.78%)	19 (42.22%)	

Having children (*N* (%))	No	22 (48.89%)	18 (40%)	0.396
	Yes	23 (51.11%)	27 (60%)	

Employment status (*N* (%))	Employed	30 (66.67%)	28 (62.22%)	0.660
	Unemployed	15 (33.33%)	17 (37.78%)	

*Note: N*, number.

**Table 2:** Comparison of GHQ-28, PHQ-15, VAS, and BI between intervention and control groups.

Criteria	Online group				Brochure group				Total	
	Pre (mean ± SD)	Post (mean ± SD)	*p*-value^1^	Effect size (95% CI^2^)	Pre (mean ± SD)	Post (mean ± SD)	*p*-value^1^	Effect size (95% CI)	*p*-value^3^	Effect size (95% CI)
GHQ-28 (total score)	58.04 ± 11.40	38.09 ± 4.04	**< 0.001**	2.05 (1.5, 2.6)	56.62 ± 11.36	39.20 ± 7.44	**< 0.001**	1.766 (1.246, 2.287)	0.258	−0.24 (−0.661, 0.18)
GHQ-28 (somatic symptoms)	18.56 ± 4.26	8.76 ± 2.44	**< 0.001**	2.863 (1.799, 3.928)	17.73 ± 3.81	8.42 ± 1.99	**< 0.001**	3.043 (2.082, 4.003)	0.634	−0.101 (−0.52, 0.319)
GHQ-28 (anxiety/insomnia)	13.13 ± 4.48	8.76 ± 2.44	**< 0.001**	1.142 (0.743, 1.542)	12.47 ± 4.67	9.11 ± 3.33	**< 0.001**	0.794 (0.494, 1.095)	0.215	−0.263 (−0.684, 0.157)
GHQ-28 (social dysfunction)	17.76 ± 4.05	13.58 ± 1.9	**< 0.001**	1.273 (0.786, 1.761)	17.6 ± 3.10	14.09 ± 1.36	**< 0.001**	1.43 (0.885, 1.974)	0.385	−0.184 (−0.604, 0.236)
GHQ-28 (depression symptoms)	8.6 ± 3.30	7.51 ± 1.56	**0.006**	0.347 (0.103, 0.59)	8.82 ± 3.18	7.58 ± 1.8	**0.005**	0.453 (0.135, 0.771)	0.782	0.058 (−0.361, 0.477)
VAS	14.86 ± 14.38	1.03 ± 2.43	**< 0.001**	1.353 (0.837 1.869)	12 ± 9.42	0.61 ± 2.19	**< 0.001**	1.609 (1.018 2.201)	0.307	−0.193 (−0.586, 0.199)
PHQ-15	12.48 ± 5.97	1.14 ± 1.92	**< 0.001**	2.528 (1.481, 3.574)	10.48 ± 5.87	1.26 ± 2.77	**< 0.001**	1.943 (1.058, 2.827)	0.211	−0.352 (−0.917, 0.213)
BI	19.78 ± 1.06	19.93 ± 0.45	0.254	−0.165 (−0.45, 0.121)	19.98 ± 0.15	20	0.323	−0.211 (−0.634, 0.213)	0.334	0.206 (−0.214, 0.626)

*Note:* 1, Analysis within groups regarding the pre- and posteducation scores; 2, 95% confidence interval; 3, analysis between groups regarding the difference between pre- and posteducation scores. Bold values indicate statistical significance.

## Data Availability

The data that support the findings of this study are available upon request from the corresponding author.

## Supporting Information

Supporting Information 1. Consort checklist for randomized controlled trials.

## References

[1] Xiao H., Zhang Y., Kong D., Li S., Yang N. (2020). Social Capital and Sleep Quality in Individuals Who Self-Isolated for 14 Days During the Coronavirus Disease 2019 (COVID-19) Outbreak in January 2020 in China. *Medical Science Monitor*.

[2] Pfefferbaum B., North C. S. (2020). Mental Health and the COVID-19 Pandemic. *New England Journal of Medicine*.

[3] Grasselli G., Pesenti A., Cecconi M. (2020). Critical Care Utilization for the COVID-19 Outbreak in Lombardy, Italy: Early Experience and Forecast During an Emergency Response. *JAMA*.

[4] Wang T. J., Chau B., Lui M., Lam G.-T., Lin N., Humbert S. (2020). Physical Medicine and Rehabilitation and Pulmonary Rehabilitation for COVID-19. *American Journal of Physical Medicine and Rehabilitation*.

[5] Rochester C. L., Vogiatzis I., Holland A. E. (2015). An Official American Thoracic Society/European Respiratory Society Policy Statement: Enhancing Implementation, Use, and Delivery of Pulmonary Rehabilitation. *American Journal of Respiratory and Critical Care Medicine*.

[6] Barker-Davies R. M., O’Sullivan O., Senaratne K. P. P. (2020). The Stanford Hall Consensus Statement for Post-COVID-19 Rehabilitation. *British Journal of Sports Medicine*.

[7] Kaminsky D. A., Guntupalli K. K., Lippmann J. (2017). Effect of Yoga Breathing (Pranayama) on Exercise Tolerance in Patients With Chronic Obstructive Pulmonary Disease: A Randomized, Controlled Trial. *Journal of Alternative & Complementary Medicine*.

[8] Holland A. E., Malaguti C., Hoffman M. (2020). Home-Based or Remote Exercise Testing in Chronic Respiratory Disease, During the COVID-19 Pandemic and Beyond: A Rapid Review. *Chronic Respiratory Disease*.

[9] Sürme Y., Özmen N., Ertürk Arik B. (2021). Fear of COVID-19 and Related Factors in Emergency Department Patients. *International Journal of Mental Health and Addiction*.

[10] Dorje T., Zhao G., Tso K. (2019). Smartphone and Social Media-Based Cardiac Rehabilitation and Secondary Prevention in China (SMART-CR/SP): A Parallel-Group, Single-Blind, Randomised Controlled Trial. *The Lancet Digital Health*.

[11] Dong X., Yi X., Gao D. (2019). The Effects of the Combined Exercise Intervention Based on Internet and Social Media Software (CEIBISMS) on Quality of Life, Muscle Strength and Cardiorespiratory Capacity in Chinese Postoperative Breast Cancer Patients: A Randomized Controlled Trial. *Health and Quality of Life Outcomes*.

[12] Li Y., Qian H., Yu K., Huang Y. (2022). The Long-Term Maintenance Effect of Remote Pulmonary Rehabilitation via Social Media in COPD: A Randomized Controlled Trial. *International Journal of Chronic Obstructive Pulmonary Disease*.

[13] Jiang Y., Liu F., Guo J. (2020). Evaluating an Intervention Program Using WeChat for Patients With Chronic Obstructive Pulmonary Disease: Randomized Controlled Trial. *Journal of Medical Internet Research*.

[14] Ames-Guerrero R. J., Barreda-Parra V. A., Huamani-Cahua J. C. (2020). Psychometric Properties and Factor Invariance for the General Health Questionnaire (GHQ-28): Study in Peruvian Population Exposed to the COVID-19 Pandemic. *ASEAN Journal of Psychiatry*.

[15] Kroenke K., Spitzer R. L., Williams J. B. (2002). The PHQ-15: Validity of a New Measure for Evaluating the Severity of Somatic Symptoms. *Psychosomatic Medicine*.

[16] Dewing J. (1992). A Critique of the Barthel Index. *British Journal of Nursing*.

[17] Bijur P. E., Silver W., Gallagher E. J. (2001). Reliability of the Visual Analog Scale for Measurement of Acute Pain. *Academic Emergency Medicine*.

[18] Rouzfarakh M., Deldar K., Froutan R., Ahmadabadi A., Mazlom S. R. (2021). The Effect of Rehabilitation Education Through Social Media on the Quality of Life in Burn Patients: A Randomized, Controlled, Clinical Trial. *BMC Medical Informatics and Decision Making*.

[19] Yilmaz M., Sahin M., Algun Z. C. (2019). Comparison of Effectiveness of the Home Exercise Program and the Home Exercise Program Taught by Physiotherapist in Knee Osteoarthritis. *Journal of Back and Musculoskeletal Rehabilitation*.

[20] Pehlivan E., Palalı İ., Atan S. G., Turan D., Çınarka H., Çetinkaya E. (2022). The Effectiveness of Post-Discharge Telerehabilitation Practices in COVID-19 Patients: Tele-COVID Study-Randomized Controlled Trial. *Annals of Thoracic Medicine*.

[21] Sunthornsup W., Vilaiyuk S., Soponkanaporn S. (2022). Effect of Educational Brochure Compared With Video on Disease-Related Knowledge in Patients With Juvenile Idiopathic Arthritis: A Randomized Controlled Trial. *Frontiers in Pediatrics*.

[22] Petkovic J., Duench S., Trawin J. (2021). Behavioural Interventions Delivered Through Interactive Social Media for Health Behaviour Change, Health Outcomes, and Health Equity in the Adult Population. *Cochrane Database of Systematic Reviews*.

[23] Farpour H. R., Hoveidaei A. H., Habibi L., Moosavi M., Farpour S. (2020). The Impact of Social Media Use on Depression in Multiple Sclerosis Patients. *Acta Neurologica Belgica*.

[24] Peretti A., Amenta F., Tayebati S. K., Nittari G., Mahdi S. S. (2017). Telerehabilitation: Review of the State-of-the-Art and Areas of Application. *JMIR Rehabilitation and Assistive Technologies*.

[25] Basirat A., Raeisi Shahraki H., Habibi L. (2020). The Correlation Between Using Social Networks and the General Health of Multiple Sclerosis Patients. *Multiple Sclerosis International*.

[26] Leochico C. F. D., Espiritu A. I., Ignacio S. D., Mojica J. A. P. (2020). Challenges to the Emergence of Telerehabilitation in a Developing Country: A Systematic Review. *Frontiers in Neurology*.

[27] Ozlu O., Atilgan E. (2024). The Effect of High-Intensity Laser Therapy on Pain and Lower Extremity Function in Patellofemoral Pain Syndrome: A Single-Blind Randomized Controlled Trial. *Lasers in Medical Science*.

[28] Hegazy F. A., Mohamed Kamel S. M., Abdelhamid A. S., Aboelnasr E. A., Elshazly M., Hassan A. M. (2021). Effect of Postoperative High Load Long Duration Inspiratory Muscle Training on Pulmonary Function and Functional Capacity after Mitral Valve Replacement Surgery: A Randomized Controlled Trial with Follow-Up. *PLoS One*.

